# Apatinib in refractory radiation-induced brain Edema: A case report: Erratum

**DOI:** 10.1097/MD.0000000000009129

**Published:** 2017-12-01

In the article, “Apatinib in refractory radiation-induced brain Edema: A case report”,^[1]^ which appeared in Volume 96, Issue 46 of *Medicine*, the authors would like to note that they received funding from Beijing Xisike Clinical Oncology Research Foundation (2017HX0008).
